# Body image dissatisfaction, nutritional status and weight control strategies among university undergraduates in Lagos: a descriptive cross-sectional study

**DOI:** 10.11604/pamj.2023.45.112.27382

**Published:** 2023-06-29

**Authors:** Foluke Adenike Olatona, Bonuola Funmilayo Aladelokun, Omolola Olayeni Adisa, Adedoyin Oyeyimika Ogunyemi, Olayinka Olufisayo Goodman

**Affiliations:** 1 Department of Community Health and Primary Care, College of Medicine, University of Lagos, Lagos State, Nigeria,; 2 Department of Community Health & Primary Care, Lagos State University College of Medicine, Lagos State, Nigeria

**Keywords:** Body image, body image dissatisfaction, nutritional status, weight management strategies

## Abstract

**Introduction:**

body image dissatisfaction has been associated with poor nutritional status and unhealthy weight management strategies. This study determined the prevalence and relationships between body image dissatisfaction, nutritional status, and weight management strategies among university undergraduate students in Lagos, Nigeria.

**Methods:**

a descriptive cross-sectional study employed a multi-stage sampling technique to select 865 undergraduates in Lagos. A pretested self-administered questionnaire was used to assess the variables. Stunkard figure rating scale was used to determine body image dissatisfaction. Body mass index (BMI) was calculated to determine nutritional status. A standard weight control strategy scale was adopted to determine weight management strategies. SPSS (version 23) was used for analysis and the association between variables was determined using Chi-square. The level of significance was set at P= <0.05.

**Results:**

the prevalence of body image dissatisfaction was high (63.5%) but not associated with gender. The majority (65.1%) had normal BMI, 10.6% were overweight and 7.2% were obese. Majority of the respondents (93.3%) engaged in weight management practices with dietary control being the most employed strategy. The most commonly employed unhealthy practice is strict dieting (37.7%). Body image dissatisfaction was significantly associated with overweight/obesity (P=0.001) but not with weight management practices. Age and overweight/obesity were predictors of BID.

**Conclusion:**

prevalence of body image dissatisfaction, overweight and obesity, and unhealthy weight management strategies were high. Body image dissatisfaction was associated with obesity but not associated with weight management strategies. All undergraduates need health education on body image and appropriate weight management strategies.

## Introduction

Body image is a multidimensional construct encompassing how one thinks, feels, and acts toward one´s body [1]. It includes a person's perception of his/her physical self and the thoughts and feelings, positive, negative or both, which result from that perception [2]. In the past, thinness was associated with illness but in the twentieth century, thinness has become an indicator of good health, while, being overweight has been associated with diseases, unattractiveness and other characteristics [3]. Generally, young people tend to be influenced by social and cultural factors that affect how they perceive their body shape and size [4]. While this may be a normal part of development, morbid situations exist in which individuals have dissatisfying perceptions of their body image [4] and this has been associated with problems with eating patterns [5,6]. Although body dissatisfaction and related consequences are thought to be a Western societal phenomenon; it has made its presence felt in developing countries [7]. Body image perception also does not always correlate with actual nutritional status of individuals; which is a more objective assessment. Previous studies on body image satisfaction reported different prevalence rates of dissatisfaction with body image; 62% in Europe [8], 48.1% in Malaysia [9], 51% in Iran [10] and 73.6% in Saudi Arabia [11]. In Nigeria, a survey reported a prevalence of 82.9% body image dissatisfaction among public and private school students in Benin, Edo State [12].

Adolescents especially girls have concerns about weight, body shape and self-image with many being dissatisfied with their body size and weight because slimness is seen as the desirable and beauty standard. Studies have shown that females usually express greater body dissatisfaction than males [13]. Poor body image perceptions can cause low self-esteem and self-confidence [14]. Some studies show that individuals who are overweight or obese are usually dissatisfied with their body image while some others have reported that the level of body image dissatisfaction is high even among those with normal body mass index values [15,16]. Obesity has been identified as one of the rising epidemics across the globe with consequential rise of non-communicable diseases incurring disproportionate health care cost on individuals, family and society. According to the latest WHO estimates, the worldwide prevalence of obesity nearly tripled between 1975 and 2016; 39% of adults (39% of men and 40% of women) were overweight in 2016, while about 13% of the world´s adult population (11% of men and 15% of women) were obese. Over 340 million children and adolescents aged 5-19 were overweight or obese in 2016 [17].

The transition from adolescence to adulthood is an important period for establishing behavioral patterns that affect long-term health and chronic disease risk [18]. Young adults who previously had little or no control over their food choices shift to having prime control over what, when and how they want to eat. Many individuals develop habits that are harmful to their health such as drinking excessive amounts of alcohol and eating unhealthily during young adulthood [19]. Apart from dietary patterns changing, young adults tend to be concerned about their physical appearance and how to change or maintain it for various reasons including appealing to romantic partners, fitting into certain peer groups or simply gaining confidence in themselves [8,20]. Body dissatisfaction among young adults influences weight control behaviours. Moreover, university undergraduates are identified as one of the populations affected by body image related issues, including body image dissatisfaction as they tend to be more concerned with their body image because of their environment [21].

Three decades ago, Nigerian university students were satisfied with their body parts [22]. However, recent studies have reported a high prevalence of body dissatisfaction and probable psychiatric morbidity among secondary school learners, with the majority (87.4%) having incorrectly perceived their actual body size [12,23]. In addition, there is a high prevalence of weight misperceptions and a lack of implementing weight control practices among adults in Northern Nigeria [24]. Exploring perceptions of individuals´ weight status and relating this perception to the real weight can help in determining the unrealistic views of body image [25]. Studies conducted among undergraduates in Abia and Edo States reported that 26.7% of students did not have an accurate perception of their body weight and the prevalence of body shape dissatisfaction was high [12,26], however, none of the published studies determined the relationship between body image perception, nutritional status and weight control strategies among university undergraduates. This study was conducted to determine the prevalence of body image dissatisfaction, nutritional status and weight management strategies as well as the relationships between these parameters among university undergraduates in Lagos, Nigeria.

## Methods

**Design of the study:** a descriptive cross sectional study design was used for the study.

**Setting and relevant dates:** the study was conducted among university undergraduate students in Lagos State. Two universities were selected from the existing three which are University of Lagos which is located in Akoka Lagos and Lagos State University which is located in Ojoo, Lagos. The participants were recruited and data was collected between August and October 2019.

**Participants:** eligibility for study was being a full-time student. Pregnant students were excluded from the study. A multistage random sampling technique was used for the study. The first stage was the selection of two universities by balloting (University of Lagos and Lagos State University) from the three universities in Lagos State. At stage 2, four (4) faculties were selected from the twelve faculties in each university using simple random sampling technique (balloting) to get eight faculties. At stage 3, three departments were selected using simple random sampling technique (balloting) from each faculty to make twenty-four departments. At Stage 4, a level was selected from each department using simple random sampling technique to obtain twenty-four classes. At the last stage, a systematic random sampling method was used to select at least thirty-three students from each class using the class list as the sampling frame. The class list was obtained, and the sample size needed from each class was used to divide the class list in order to determine the sampling interval in each class. Students were then recruited based on the sample size and sampling interval from each class. Balloting was used to determine the first out of the first ten students on the class list.

**Variables:** the independent variable was body mass index while the outcome variables were body image dissatisfaction and weight management strategies.

**Data sources and measurement:** data was collected using semi-structured, self-administered questionnaire and pro-forma for recording anthropometry. Pre-test was done among thirty full-time undergraduate students of Caleb University with a view to testing the reliability of the instrument and making appropriate corrections, where necessary. The participants were told to define weight management as activities or lifestyle changes that were done to control weight gain or loss or that were done to maintain a certain body weight. The first section of the questionnaire on socio-demographic characteristics was adapted from past literature review [25,27]. The second section consists of the Stunkard figure rating scale (FRS), for measuring body dissatisfaction [28]. The scale consists of silhouette drawings ranging from extremely thin (1) to very obese (9) in appearance. From the 9 body figures, participants were asked to identify their perceived body image (i.e., how they think they look) and ideal body image (i.e., what they want to look like). The third section consists of the weight control strategy scale (WCSS), a validated self-report instrument developed by Pinto *et al*., used to assess weight control behaviors. The 30-item WCSS contains 4 subscales: Dietary Choices, Self-monitoring Strategies, Physical Activity, and Psychological Coping. Each item is rated on a Likert scale from 0 (never) to 4 (always) [29]. The anthropometric measurements, i.e., height and weight of each student were taken according to the standard technique as described by the World Health Organization to assess the nutritional status of the students [30].

**Bias: r**eported weight management strategies might not have been the actual weight management strategies in practice. Reporting or social acceptability bias might have been introduced.

**Study size:** the minimum sample size calculated using the Cochran´s formula (no= Z^2^pq/e^2^) was 470 (where p was 54.8%; the prevalence of body image dissatisfaction in a previous study [21] but a higher size of 865 was used.

**Quantitative variables:** statistical analysis was done using Statistical Package for Social Sciences (SPSS Version 23). Socio-demographic data was analyzed using descriptive statistics. Frequency distribution was generated for variables. Body image perception was analyzed using the scores on the Stunkard figure rating scale. The difference between perceived body image and desired body image was used to determine the level of dissatisfaction with current body image. Values other than zero represented body image dissatisfaction. A positive value is indicative of the participant´s wish to be thinner than the perceived current size, while a negative value reflects the participant´s wish to be heavier than the current perceived size [28]. Weight management strategies were analyzed using the scores on the weight control strategies scale. All item scores were added and divided by 30 to get the total score. To obtain the subscale score, each subscale item scores was summed up and divided by the number of items in the subscale as follows: Dietary control (10 items): 19, 20, 21, 22, 23, 24, 25, 26, 27, 28; Self-monitoring strategies (7 items): 29, 30, 31, 32, 33, 34, 35; Physical activity (6 items): 36, 37, 38, 39, 40, 41; Psychological coping (7 items): 42, 43, 44, 45, 46, 47, 48; Higher scores indicated greater use of weight management strategies. The body mass index (BMI) of the respondents was calculated using the weight and height by dividing the weight in kilograms by the height in meters squared (weight/height2). The BMI was analyzed according to WHO standards and categorized as follows: underweight (BMI < 18.5), normal weight (BMI 18.5-24.9), overweight (BMI 25-29.9), and obese (BMI > 30).

**Statistical methods:** associations between variables were calculated using Chi-square (χ2) test with level of significance set at p =< 0.05.

**Ethical consideration:** ethics approval was obtained from the Human Research and Ethics Committee of the Lagos University Teaching Hospital and Lagos State University Teaching Hospital. The approval numbers are ADM/DCST/HREC/APP/3073 and LREC/06/10/1296 respectively. The research was conducted according to the principles expressed in the Declaration of Helsinki. Permission was obtained from the dean of students´ affairs of the two schools, and informed written consent was obtained from each respondent before participating in the research. Confidentiality was assured and maintained throughout the study.

## Results

**Figure 1:**
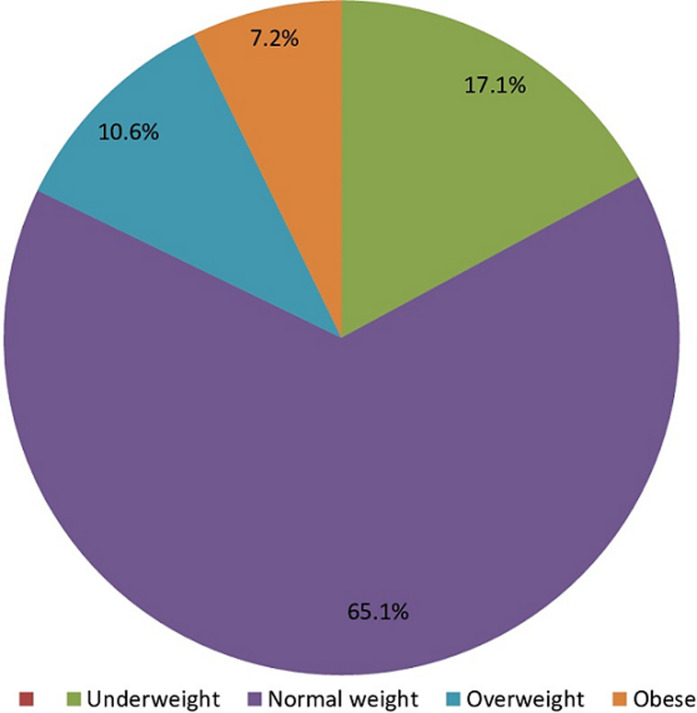
nutrition status of university undergraduate students in Lagos, Nigeria

**Descriptive data:** the mean age was 20.68±2.96 years. Age ranged from 15 to 31 years with a higher proportion (59.9%) in the 15-20 years age group. There were 467 female students (54.0%) and 398 male students (46.0%) who participated in the study. Majority of the respondents were Christians (71.1%) and 93.8% were single. Most of them were within the normal BMI range. The prevalence of underweight was 17.1%, overweight 10.6% while 7.2% were obese (Figure 1).

**Outcome data:** the prevalence of body image dissatisfaction was 63.5% among the students. The prevalence of dissatisfaction was not associated with gender (P=0.445), however, the pattern of dissatisfaction showed that more males desired to be heavier while more female students desired to be thinner (Table 1). Majority of the respondents, 807 (93.3%) engaged in weight management practices. Healthy dietary control was the most frequent strategy employed (39.4%); only 19.6% of the respondents engaged in physical activity, and 12.5% used combination of one or more of the methods. The most commonly employed unhealthy practices are strict dieting (37.7%) and use of herbal mixtures (37.5%) (Table 2).

**Table 1:** prevalence of body image dissatisfaction and pattern of dissatisfaction according to gender

	Body Image dissatisfaction (n=865)		Pattern of dissatisfaction (n=549)		Total
	Satisfied (%)	Dissatisfied (%)	Desire to be thinner	Desire to be heavier	
Female	176 (37.7)	291 (62.3)	134 (28.7)	157 (33.6)	467 (100.0)
Male	140 (35.2)	258 (64.8)	89 (22.4)	169 (42.5)	398 (100.0)
Total	316 (36.5)	549 (63.5)	223 (25.8)	326 (37.7)	865 (100.0)

X2: 0.585; P = 0.445; X2: 7.566; P = 0.006

**Table 2:** weight management strategies employed by the respondents

Strategy	Freq	Percentage (%)
	(n=807)	
Dietary control	318	39.4
Physical activity	158	19.6
Psychological coping	149	18.5
Self- monitoring strategy	81	10
Dietary control & Physical activity	20	2.5
Dietary control & Psychological coping	15	1.9
Dietary control &Self-monitoring strategy	7	0.9
Physical activity & Psychological coping	14	1.7
Self- monitoring strategy & Physical activity	8	1
Self- monitoring strategy & Psychological coping	11	1.4
Self - monitoring strategy, Physical activity & Psychological coping	5	0.6
Dietary control, Self-monitoring strategy, Physical activity & Psychological coping	21	2.6
**Others**		
Use of diet pills (supplements, Appetite suppressants)	277	34.3
Use of herbal mixtures	303	37.5
Self-induced vomiting	178	22.1
Use of methamphetamine (“tik”)	138	17.1
Use of laxatives	159	19.7
Use of diuretics	148	18.3
Smoking more cigarettes	95	11.8
Strict dieting	304	37.7
Adequate water intake	240	29.4
Adequate duration of sleep	573	71.0

The weight management strategy employed by the students was not significantly associated with the level of dissatisfaction with their body image (P=0.095) as many students used weight management strategies regardless of their body image dissatisfaction. The prevalence of body image dissatisfaction was found to be high across all BMI groups. The highest dissatisfaction (89.7%) was among the obese group, (P=0.001). However, there was no statistically significant association between nutritional status and weight management strategies, P=0.087 (Table 3). Only age and overweight/obesity were predictors of body image dissatisfaction. Undergraduates between age 15 and 20 years were 2.25 times more dissatisfied with their body image compared to others (p=0.013). Overweight and obese undergraduates were also 0.24 times more dissatisfied with their body image compared to those who had normal weight, (P=0.016) (Table 4).

**Table 3:** association between body image dissatisfaction and body mass index/weight management strategies

Body image Dissatisfaction	Body Mass Index					χ2	P-Value
	**Under weight**	**Normal**	**Over weight**	**Obese**	**Total**		
Satisfied	44 (32.8)	205(39.0)	31 (34.8)	6 (10.3)	286(35.4)	19.246	0.001
Dissatisfied	90 (67.2)	321(61.0)	58 (65.2)	52 (89.7)	521(64.6)		
	**Weight management strategies**						
	**Dietary Control**	**Physical activity**	**Psychological coping**	**Self-monitoring strategy**	**Total**		
Satisfied	129(51.0)	51 (20.2)	52 (20.6)	21 (8.3)	253(100.0)	7.904	0.095
Dissatisfied	189 (41.7)	107 (23.6)	97 (21.4)	60 (13.2)	453 (100.0)		

**Table 4:** predictors of body image dissatisfaction among the respondents

Parameter	Adjusted Odd’s Ratio	P value	95% Confidence interval for AOR	
			Lower Limit	Upper Limit
**Age group**				
20-31 years (Ref)	1			
15-20 years	2.251	0.013*	1.189	4.261
**Gender**				
Male (Ref)	1			
Female	1.48	0.193	0.82	2.67
**Nutritional status**				
Normal	0			
Underweight	0.513	0.122	0.22	1.195
Overweight/Obesity	0.238	0.016*	0.704	0.762
**Marital status**				
Singles	1			
Married	0.704	0.609	0.184	2.695
**Tribe /Ethnicity**				
Yoruba	1			
Others	0.97	0.844	0.715	1.316

* p value= <0.05

## Discussion

The prevalence of body image dissatisfaction in this study was 63.5%. This high prevalence is in keeping with other studies conducted around the world such as Europe (62.0%) [8] Malaysia [9] (77.8%), and Saudi Arabia (73.6%) [11]. The high prevalence of body image dissatisfaction in general could be attributed to a global social construction of standards of beauty wherein slimness is considered synonymous to beauty. This identical ideology across peoples and continents could be attributed to the inter-connectedness through social media where information is spread rapidly at the click of a button regardless of geographic and cultural boundaries. However, there was no statistically significant difference in the level of dissatisfaction with body image in the male and female genders. This finding requires further exploration as some studies in Iran and Brazil found the female students to be more dissatisfied with their body image than the males [31-33] while other studies have shown that women are much more likely than men to categorize themselves as overweight and express dissatisfaction [9,10,34,35]. A Canadian study found a strong predictor of male body image dissatisfaction to be weight-height ratio [36]. The disparity seen in this study may be adduced to differences in societal expectations between developed and developing countries which may influence satisfaction levels.

Most of the participants in this study employed weight management strategies irrespective of body image satisfaction or dissatisfaction. The pattern of dissatisfaction showed that more males desired to be heavier while more females desired to be thinner. A similar trend was reported in a study conducted in Malaysia [9]. This could be in conformity to society´s perception of female attractiveness as thinness [37] as well as males´ need to appear muscular to be seen as more masculine [38]. Dietary control was the most frequent strategy used as a weight management strategy in this study while self-monitoring was the least used. Studies have shown that diet control is a critical component of weight management strategies [39].

Most of students were within the normal BMI range which correlates with previous studies conducted among undergraduate students in Nigeria [40,41], however, the majority 807 (93.3%) engaged in weight management practices. Females were more involved in weight management practices than males. This trend was also reported in a study conducted in Malaysia 9 and another study in Mauritius, which found majority of the respondents involved in weight control practices to be females [42]. The weight management strategies identified among the students in this study were dietary control, physical activity, psychological coping and self-monitoring strategies. This finding was similar to a study in the United States where strategies included weighing self, increasing exercise intensity and increasing intake of fruits [43]. In Ghana, physical activities, lifestyle modifications and active dieting were the strategies employed while in UAE dietary control and physical activity were employed [25,44]. The students engaged in unhealthy management strategies such as strict dieting more than the healthy strategies. Unhealthy strategies included eating little food, skipping meals, fasting, smoking more cigarettes, self-induced vomiting, use of laxatives, diet pills and herbal mixtures were also identified among university undergraduate students in South Africa 35. The high prevalence of the use of weight control strategies especially unhealthy strategies shows that university undergraduates do not have robust knowledge of healthy weight management strategies and that there is a need to provide weight management education to them.

Age was a predictor of body image dissatisfaction. Undergraduates in the age group 15-20 years were 2.25 times more dissatisfied with their body image compared to the older ones, (p=0.013). This finding correlates with a previous study which identified age as a predictor of BID [32] but another study among undergraduates in UAE did not reveal any significant difference for BID among the four age groups [45]. More studies would be needed in the future to explore the role of age. Nutrition status was statistically significantly associated with body image dissatisfaction (P=0.001). This agrees with previous research which found that study participants who were overweight were more dissatisfied compared with those who had normal weight [32]. On the other hand, another research showed that those who expressed body image dissatisfaction were 70% less likely to have a healthy nutrition status. Studies among Moroccan university undergraduate students showed that those who were underweight and overweight had significantly higher prevalence of dissatisfaction compared to those who had normal weight [46].

This current study confirms that overweight/obesity is a predictor of BID. Undergraduate students who were overweight/obese were 0.24 times more dissatisfied with their body image compared to those who had normal weight. Another study in Arabia revealed that BMI was a predictor for dissatisfaction [47]. Significant correlation has also been reported in previous studies [48,49]. There was no significant association between body image dissatisfaction and weight management practices. This is similar to the findings in a Ghanaian study [25], but contrary to another study in Malaysia where it was shown that body image dissatisfaction was significantly associated with dietary control [9]. This study also did not demonstrate any association between nutritional status and weight management strategies.

**Limitation:** a cross-sectional study was used for this study and would only allow a measure of association. A longitudinal study design would have been better in obtaining strengthened inferences of causality. The weight management strategy scale used to assess the weight management strategies might not have measured actual strategies employed but merely the reported strategy. A different study design and statistical methods would have been required to ascertain actual practice of these measures, being aware of biases (reporting/social acceptability bias etc.).

## Conclusion

Body image dissatisfaction was high and associated with obesity but did not influence weight management practices among university undergraduate students in Lagos. The study can be applied to all undergraduates in Lagos State. The findings from this study call attention to the need to provide education to university undergraduate students on healthy weight management practices.

### What is known about this topic

- *The prevalence of body image dissatisfaction is high among university undergraduates;*
- *Body image dissatisfaction is associated with nutritional status and weight management strategies*.

### What this study adds

- *There is high uptake of reported weight management strategies among university undergraduates in Lagos;*
- *Undergraduate students who were obese had higher prevalence of body image dissatisfaction in Lagos, Nigeria;*
- *Body image dissatisfaction is not associated with weight management strategies among university undergraduates in Lagos*.
